# Applications and Properties of Magnetic Nanoparticles

**DOI:** 10.3390/nano11051297

**Published:** 2021-05-14

**Authors:** Paolo Arosio

**Affiliations:** 1 Dipartimento di Fisica and INFN, Università degli Studi di Milano, Via Celoria 16, 20133 Milan, Italy; paolo.arosio@unimi.it; 2 Consorzio Interuniversitario Nazionale per la Scienza e Tecnologia dei Materiali–Milano Unit, Via Celoria 16, 20133 Milan, Italy; 3 INFN, Istituto Nazionale di Fisica Nucleare–Milano Unit, Via Celoria 16, 20133 Milan, Italy

In the last few decades, magnetic nanoconstructs have attracted increasing attention due to, among others, their specific magnetic properties and huge number of applications in completely different fields. The purpose of this Special Issue is to cover the new developments in synthesis, characterization, and fundamental studies of magnetic nanoconstructs, ranging from conventional metal oxide nanoparticles, to novel molecule-based or hybrid multifunctional nano-objects. At the same time, this Special Issue is intended to focus on and explore the potential of these novel magnetic nanoconstructs in nanomedicine and biology, energy harvesting and storage applications, sensing applications, pollution remediation, data storage, and several other possible applications.

Without claiming to be exhaustive in such a broad research field, this Special Issue provides at least a partial snapshot of the state-of-the-art magnetic nanomaterial studies, showing several recent advances, new ideas, and open problems.

The collection includes 17 research articles by authors from 18 countries, all over the world, which shows once more the global scientific interest in the magnetism of nano-objects and its crucial role in many new technological areas.

The original articles of this Special Issue cover several scientific themes and arguments associated with synthesis, characterization, and the use of magnetic nanoconstructs. The topics covered by the collection are summarized here below.

Engelmann et al. studied a dual frequency magnetic excitation of magnetic nanoparticles for biosensing applications [1], comparing their experimental results with a thermal equilibrium model, based on Langevin function and a micromagnetic Monte-Carlo (MC)-simulation method that models the non-equilibrium dynamics of nanoparticles.

Cheon et al. [2] proposed new magnetic nanoparticles, functionalized with histidine as nano-enzymes for the detection of acetylcholine, with high specificity and sensitivity.

In Ref. [3] Omelyanchik and co-workers focused on the preparation and study of polymer-based nanocomposites that include magnetic nanoparticles with intriguing piezoelectric properties. The obtained results suggested a possible future application of these magnetoelectric composites as bioactive surfaces for guiding the differentiation of neuronal stem cells.

Hydrophilic magnesium iron oxide nanoparticles, presented by Darwish et al. exhibited high heating efficiency as hyperthermic system and a good Magnetic Particle Imaging (MPI) signal with adequate spatial resolution [4]. Bringing together these physical properties, the proposed magnetic nanomaterials could be interesting for various biomedical applications, such as image-guided cancer treatment.

Petrichenko and co-workers produced magnetoliposomes starting from 1,4-dihydropyridines with different substituents and demonstrated that the properties of liposomes did not change varying the length of alkyl chain or changing substituents at position 4 of 1,4- dihydropyridines [5]. 

Novickij et al. used nisin-loaded magnetic nanoparticles stimulated by an alternating magnetic field as a method for the treatment of drug-resistant foodborne pathogens and, in particular, they showed its efficacy to decrease the resistance to the drug of *Listeria innocua* bacteria [6]. 

The immobilization of porcine pancreatic alpha-trypsin (PPT) by means of molecularly imprinted polymers, fabricated on magnetic nanoparticles, was obtained by Kanubaddi et al. The authors demonstrated that immobilized enzyme maintains its catalytic activity also after several cycles of use, paving the way for a new method of point immobilization of enzymes [7]. 

Co/Cu multi-layered nanocylinders, electrodeposited into anodized aluminium oxide nanochannels, were synthesized and characterized by Mizoguchi and co-workers [8]. The nanocylinders showed high current-in-plane giant magnetoresistance performances and a cobalt spin–diffusion length that can be described by Valet–Fert equation. 

Fuhrmann et al. proposed a novel approach for the magnetic imaging of superparamagnetic nanoparticles encapsulated in a polymer matrix, using data fusion of Magnetic Force and Atomic Force Microscopies [9]. The numerical method developed by authors allowed us to minimize topographic crosstalk and consequently, to characterize local magnetic properties of nanoparticles. 

Meanwhile, Brero and co-workers discussed the effect of negative polyelectrolyte coatings on the magnetic resonance properties of maghemite nanoparticles with two core dimensions [10]. The authors stated that the chosen nanoparticles are promising superparamagnetic T_2_ contrast agents for MRI, but the polyelectrolyte coatings poorly influenced their longitudinal and transverse relaxometric properties. 

In Ref. [11], Usov and Gubanova used a theoretical approach based on the stochastic Landau–Lifshitz equation to investigate which are the optimal physical characteristics of magnetosome chains, used as heat mediator in magnetic hyperthermia. 

Room-temperature magnetic memory effect from Fe-doped NiO nanoparticles, synthesized and characterized by Gandhi et al. was correlated to the formation of defect clusters that enhanced intraparticle interactions. Consequently, the magnetic anisotropy of the nanoparticles changed, causing a different behaviour of magnetic moments relaxation processes that could be intriguing for the design of future spintronic devices [12]. 

Omelyanchik et al. deeply studied the magnetic properties of nanocomposites consisting of Co–ferrite nanoparticles, dispersed in a SiO_2_ matrix. The results presented by authors showed that the annealing temperature changed the magnetocrystalline anisotropy and the saturation magnetization of the nanocomposite, modifying the particle size and also influencing the magnetic disorder of their surface [13]. 

Furthermore, Dutz and co-workers presented Co-doped iron oxide nanoparticles with tunable magnetic properties. The most promising particle–magnetic field combination seemed to be suitable for application in magnetic fluid hyperthermia, given that cell viability analysis also did not show an increased toxicity for the Co-doped particles, compared to the pure iron oxide ones [14]. 

Vangijzegem et al. proposed a continuous flow system preparation of very small iron oxide nanoparticles by thermal decomposition. Studying the influence of experimental parameters (e.g., ligand concentration and nature, temperature, etc.) on the nanoparticles magneto-crystalline and relaxometric properties, the authors showed that they had incidence on relaxometric and magnetic properties, while they did not affect size properties of the nanoparticles [15]. 

Darwish et al. reported interesting results on magnetic ferrite nanoparticles, with high performances again for the magnetic hyperthermia “on fire” topic [16]. In particular, they compared cobalt–ferrite (8 ± 2 nm) and zinc–cobalt ferrite (25 ± 5 nm) nanoparticles coated with sodium citrate, evaluating their heat release, and testing their cytocompatibility.

In the last article, Chen et al. developed a plasmonic vector magnetometer, based on a side-polished few-mode-fiber, functionalized by MNPs [17]. The optical-fiber magnetic sensor exhibited high sensitivity to both the magnetic-field intensity and orientation. Therefore, considering its compact size and online detection scheme, it could be used in the future for the detection of weak magnetic-field vectors.

In conclusion, this Special Issue of *Nanomaterials* collects a series of original research articles of front-line researchers, with an interdisciplinary approach on exploring the use of magnetic nano-objects in a broad range of applications. I am confident that this Special Issue will provide the reader with an overall view of the latest prospects in this fast evolving and cross-disciplinary field.
